# Enhanced fatty acid methyl esters recovery through a simple and rapid direct transesterification of freshly harvested biomass of *Chlorella vulgaris* and *Messastrum gracile*

**DOI:** 10.1038/s41598-021-81609-6

**Published:** 2021-02-01

**Authors:** Saw Hong Loh, Mee Kee Chen, Nur Syazana Fauzi, Ahmad Aziz, Thye San Cha

**Affiliations:** 1 Faculty of Science and Marine Environment, Universiti Malaysia Terengganu, 21030 Kuala Terengganu, Terengganu Malaysia; 2 Satreps-Cosmos Laboratory, Central Laboratory Complex, Universiti Malaysia Terengganu, 21030 Kuala Terengganu, Terengganu Malaysia; 3 Institute of Marine Biotechnology, Universiti Malaysia Terengganu, 21030 Kuala Terengganu, Terengganu Malaysia

**Keywords:** Gas chromatography, Fatty acids, Oils, Biodiesel

## Abstract

Conventional microalgae oil extraction applies physicochemical destruction of dry cell biomass prior to transesterification process to produce fatty acid methyl esters (FAMEs). This report presents a simple and rapid direct transesterification (DT) method for FAMEs production and fatty acid profiling of microalgae using freshly harvested biomass. Results revealed that the FAMEs recovered from *Chlorella vulgaris* were 50.1 and 68.3 mg with conventional oil-extraction-transesterification (OET) and DT method, respectively. While for *Messastrum gracile*, the FAMEs recovered, were 49.9 and 76.3 mg, respectively with OET and DT methods. This demonstrated that the DT method increased FAMEs recovery by 36.4% and 53.0% from *C. vulgaris* and *M. gracile*, respectively, as compared to OET method. Additionally, the DT method recovered a significantly higher amount of palmitic (C16:0) and stearic (C18:0) acids from both species, which indicated the important role of these fatty acids in the membranes of cells and organelles. The DT method performed very well using a small volume (5 mL) of fresh biomass coupled with a shorter reaction time (~ 15 min), thus making real-time monitoring of FAMEs and fatty acid accumulation in microalgae culture feasible.

## Introduction

The key processes involved in the fatty acid profiling of microalgae are cultivation, biomass harvesting and drying, oil extraction and transesterification to produce fatty acid methyl esters (FAMEs). Among these processes, biomass drying and oil extraction are two costly and tedious processes. The conventional microalgae oil extraction method require several steps. The efficiency of the oil extraction depends on microalgae species, the cell wall disruption method and solvent system^1, 2^. To disrupt microalgae cell walls, various approaches such as grinding, ultra sonication^1^, microwave treatments^3^, enzymes digestion^4^ autoclaving, bead beating and high salt solution^5^ have been reported. The major problems faced by the conventional oil extraction-transesterification (OET) method are biomass drying and oil extraction processes. The drying of fresh/wet biomass prior to oil extraction requires high energy that is time-consuming. It is estimated that over 90% processed energy is used during the biomass drying and oil extraction steps^6, 7^. Furthermore, the solvent system cannot completely extract the fatty acids, especially the fatty acids which are attached to the membrane lipids such as phospholipids and glycolipids^8^. This leads to lower FAMEs recovery and compromises the fatty acid profiling^6, 7^. In addition, the oil extraction yield is adversely affected when wet biomass is used^9^.

Over the years, direct transesterification (DT) of biomass has received enormous attention due to its high efficiency in the reduction of energy and time^10^. The DT method enables simultaneous extraction of lipid and FAMEs conversion processes by alcohol in the presence of a catalyst. The alcohol acts as an extraction solvent and an acyl acceptor for transesterification^11–13^. The DT method makes it possible to produce FAMEs in a single step using microalgae fresh/wet biomass while also eliminating both biomass drying and oil extraction processes^7, 14^. There are plenty of recent research on DT of microalgae biomass along with relatively higher FAMEs yield reported. There are DT methods established using dry microalgal biomass^15–17^ or in combination with novel approaches such as microwave irradiation^18^, ultrasound treatment^19^ and vortex fluidic device (VFD)-based^20^, as well as the application of immobilized lipases^19, 21^, among others. One of the major drawback of the above-mentioned DT methods was the transesterification reaction time which was relatively longer and differs greatly depending on the approaches used that may range from less than one hour to several hours^15–24^. In addition, a large amount of dry/wet microalgal biomass was needed for the transesterification reaction^15–24^.

Nevertheless, there is a lack of research reported on small-scale DT using fresh/wet microalgal biomass directly harvested from microalgal cultures for real-time monitoring of oil accumulation and fatty acid composition. Therefore, this study compared the efficiency of the conventional OET and DT methods^25^ for total FAMEs recovery from small amount of microalgae biomass that was freshly and directly harvested from microalgae cultures. In this report, the DT method was optimized using different small sample volume sizes of freshly harvested microalgae biomass. The variation of fatty acid composition from the two methods was also compared.

## Results and discussion

### Cell growth, biomass production and oil content using conventional oil extraction-transesterification (OET) method

**Figure 1:**
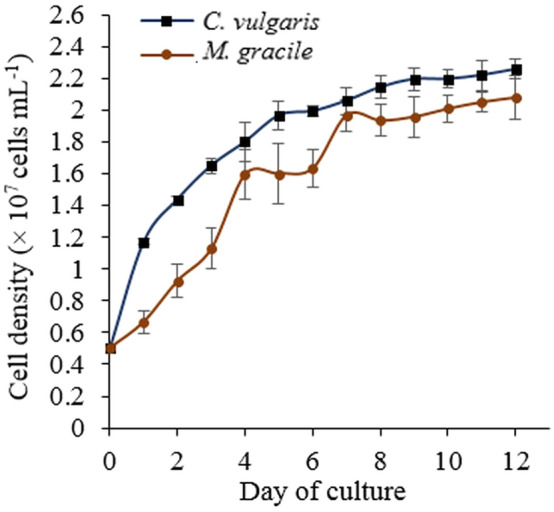
The growth curve of *C. vulgaris* UMT-M1 and *M. gracile* SE-MC4 cultured under nitrate deficient condition. The cultures were harvested at stationary growth phase when cell growth became plateau. Data shown as the mean ± SD, n = 3.

Figure 1 shows the growth curve of *C. vulgaris* and *M. gracile*. The cell density of the *C. vulgaris* culture increased exponentially in the first 5 days of the culture and attained early stationary growth phase at day 8. No significant change in cell density was observed until day 12 where the cell density of 2.26 × 10^7^ cells mL^−1^ was recorded. On the other hand, the exponential growth of *M. gracile* lasted for 7 days and attained early stationary growth phase at day 8, where no significant change in cell density was observed until day 12 with cell density of 2.08 × 10^7^ cells mL^−1^. Although an equal initial cell density (5 × 10^6^ cells mL^−1^) was used for both species, the cell densities recorded everyday were different between the two species with *C. vulgaris* consistently recording higher cell density throughout the growth curve (Fig. 1). All cultures were harvested on day 12 where cell growth reached a plateau, which represents the stationary growth phase. The logistic growth pattern of microalgae involves lag phase, log phase, stationary phase and senescence phase^26^. In the lag phase, which was at the beginning of the culture, both species were adapting to a new culture environment. After full adaptation to the new culture condition, the log phase occurred in which the population growth was greatly increased until around day 7 (Fig. 1). When the cultures reached the stationary growth phase, the nutrients in the culture medium were expected to be depleted and cell growth turned into a regression straight line with no significant change in cell density at the end of the growth curve (Fig. 1).

Generally, microalgae grow slowly and attain low biomass production, but with higher lipid^25–27^ and carbohydrate^28, 29^ production when culture is cultivated under low nitrate concentration. Furthermore, stationary growth phase has been recognized as a period of active oil accumulation phase for microalgae species due to the conversion of carbohydrate to lipids^30–32^. Therefore, both *C. vulgaris* and *M. gracile* cultures were harvested at the stationary growth phase to ensure high oil accumulation for further analysis. As shown in Table 1, the total oil content of *C. vulgaris* and *M. gracile* at the stationary growth phase was 47.5% and 40.6% (percent of dry weight), respectively. Conversely, *M. gracile* recorded higher dry biomass production of 0.42 g L^−1^ against 0.37 g L^−1^ produced by *C. vulgaris* (Table 1). The higher biomass production was concomitant with lower cell density and total oil content recorded by *M. gracile* versus *C. vulgaris*. The larger cell size of *M. gracile* could compromise the efficiency of biological and physiological processes^33, 34^.

**Table 1:** Cell density, total biomass and oil content of *C. vulgaris* and *M. gracile* harvested at stationary growth phase from 0.3 L cultures.

Species	Cell density (× 10^–7^ cells mL^−1^)	Total biomass (mg DW)	Biomass production (g DW L^−1^)	Oil content (% of DW)
*C. vulgaris*	2.26 ± 0.06	112.1 ± 5.0	0.37 ± 0.02	47.5 ± 2.4
*M. gracile*	2.08 ± 0.14	126.3 ± 2.1	0.42 ± 0.01	40.6 ± 1.4

Data shown as the mean ± SD (n = 3).

### OET versus DT for determination of total FAMEs

The DT procedure was similar to the transesterification procedure in the OET method, except the oil sample, which was replaced with the fresh/wet microalgae biomass that was directly harvested from the stationary growth phase microalgal culture. Sample aliquots ranging from 5 to 30 mL were directly sampled from *C. vulgaris* cultures for DT to determine the total FAMEs*.* The good linearity (Supplementary Fig. S1) with correlation coefficient, *r* in the range of 0.9880–0.9913 was obtained from the calibration experiment and the linearity plots were then applied to quantitate the total FAMEs in the samples. Table 2 summarizes the total FAMEs obtained from different sample volumes of *C. vulgaris* cultures using the DT method. Generally, the results showed that the smaller sample volume used in DT produced higher total FAMEs. The 5-mL sample volume produced 68.3 mg (60.9% DW) of the total FAMEs, which was significantly higher (*p* < 0.05) than the total FAMEs obtained from the 20- and 30-mL sample volumes. Interestingly, the DT method using 5 mL sample volume managed to recover 36.4% more FAMEs when compared to the OET method, in which OET produced only 50.1 mg of total FAMEs (Table 2). In view of the higher total FAMEs yield from DT using 5 mL sample volume from *C. vulgaris* culture, therefore, the 5-mL sample volume was also chosen for the analysis of *M. gracile*. As shown in Table 2, the DT method using 5-mL sample volume managed to recover 76.3 mg (or 60.4% DW) total FAMEs from *M. gracile*, which was an increment of 53.0% (or 1.53-fold) compared to 49.9 mg (or 39.4% DW) obtained with the OET method (Table 2). The calculation methods for total FAMEs as outlined in this report was proven reliable and accurate when the total FAMEs calculated from OET method was expressed in percent of dry weight (% DW). For instance, the total FAMEs of 44.7% (% DW, Table 2) calculated from the OET method and the total oil content of 47.5% (% DW, Table 1) for *C. vulgaris* were not significantly different statistically. A similar result was observed for *M. gracile* (Tables 1 and 2).

**Table 2:** Total FAME obtained from *C. vulgaris* and *M. gracile* using oil-extraction transesterification (OET) and direct transesterification (DT) methods from 0.3 L culture.

	OET method	DT method (sample volume)			
		5 mL	10 mL	20 mL	30 mL
***C. vulgaris***					
Total FAME (mg)	50.1 ± 2.9^b^	68.3 ± 5.5^a^	62.3 ± 5.9^ab^	53.2 ± 1.1^b^	49.5 ± 4.5^b^
Total FAME (% DW)	44.7 ± 2.6^b^	60.9 ± 4.9^a^	55.6 ± 5.3^ab^	47.4 ± 1.0^b^	44.1 ± 4.0^b^
***M. gracile***					
Total FAME (mg)	49.9 ± 2.8^b^	76.3 ± 9.2^a^	^−^	^−^	^−^
Total FAME (% DW)	39.5 ± 2.2^b^	60.4 ± 7.3^a^	^−^	^−^	^−^

For DT method, fresh cells were harvested from 5, 10, 20, and 30 mL of stationary phase microalgal cultures.

The values are in mean ± SD (n = 3). Different letters in the same row indicate values are significantly different according to one-way ANOVA at *p* < 0.05.

Both *C. vulgaris* and *M. gracile* varied in cell sizes. The size of *C. vulgaris* cell was in the range of 2–10 μm in diameter with a spherical shape^24^ whereas the *M. gracile* was in the range of 13–16 μm in length and 1.7–3.5 μm in width with elongated, spindle-shaped, curved with pointed ends^3, 34^. The larger *M. gracile* cell including its organelles could consist of relatively greater double-layer membranes, which is embedded with phospholipids, glycolipids and other compositions^8^. In general, microalgal cell membranes vary in thickness depending on species and stages of growth^33^. When performing DT, a greater amount of fatty acids contained in the cell membrane of *M. gracile* were transesterified into FAMEs comparing to *C. vulgaris.* Furthermore, the total FAMEs obtained from *M. gracile* through DT method showed an increment of 53.0% against OET method (Table 2). On the other hand, the total FAMEs obtained from *C. vulgaris* through the DT method showed an increment of only 36.4% against the OET method (Table 2). These results revealed that the 5-mL sample volume withdrawn directly from the microalgal cultures for DT analysis was appropriate for real-time monitoring of FAMEs production in different microalgae species.

The result demonstrated that the DT method transesterified more fatty acid than the OET method from a single cell. According to Dong et al.^35^, the alkali catalyst might convert the lipid within the cell into cell wall-permeable form or it may make the cell wall permeable to lipids. Therefore, this would enable more or entire lipids to be transesterified into FAMEs from a single cell. Moreover, DT performed on the residual biomass, which had undergone solvent extraction revealed that the total fatty acid extracted and the fatty acid remaining in the residual biomass was similar to the total FAMEs obtained using the DT method^11^. This indicated the lipid extraction process did not extract all the fatty acids. A comparative study of several OET and DT methods by Cavonius et al.^13^ concluded that the total FAMEs recovered among the DT methods was observably similar or slightly higher than the OET methods for several microalgae species. On the other hand, Wahlen et al.^12^ reported FAMEs recovery between 39 and 82% of extractible lipid from several microalgae and cyanobacteria species. In contrast, the DT method used in this current research managed to recover far more FAMEs from the fresh microalgal biomass. The 36.4 and 53% increment of total FAMEs obtained from *C. vulgaris* and *M. gracile*, respectively, using the DT method indicated that there was far more other lipid classes composed in the cell structure (in particular the membrane of cells and organelles) which was not extractable using the OET method.

### OET versus DT for determination of fatty acid composition

Table 3 shows the fatty acid composition obtained from *C. vulgaris* using the DT method. The six major fatty acids, the palmitic (C16:0), stearic (C18:0), oleic (C18:1), linoleic (C18:2), γ-linolenic (C18:3n6) and α-linolenic (C18:3n3) acids, which together contributed over 98.0% of total fatty acids were analyzed. The analysis of the fatty acid composition revealed that DT using different sample volumes did not have any effect on the composition of C16:0, C18:1, C18:3n6 and C18:3n3 (Table 3). The C16:0 was the most abundant fatty acid with composition ranging from 42.2 to 46.7% (percent of total FAMEs), followed by C18:1 with composition ranging from 17.8 to 24.0%. In the meantime, C18:3n6 and C18:3n3 contributed relatively lower amounts of 4.3–4.5% and 3.3–4.4%, respectively (Table 3). On the other hand, DT using 5-mL sample volumes rendered higher (*p* < 0.05) C18:0 (11.9%) and lower (*p* < 0.05) C18:2 (13.0%) as compared to larger sample volumes (Table 3). A minimum sample volume was always preferred in DT analysis in order to keep the real-time monitoring of FAMEs production easy. However, a sufficient sample size was important for homogeneously representing the microalgal culture biomass, as the FAMEs recovered must exceed the instrument detection limits to minimize the systematic errors. The relatively consistent fatty acid composition coupled with far more total FAMEs recovery concluded that the 5-mL sample volume was selected for subsequent comparison with the OET method.

**Table 3:** Fatty acid composition of *C. vulgaris* UMT-M1 obtained from direct-transesterification (DT) methods.

Fatty acid	DT method (% of total FAME)			
	5 mL	10 mL	20 mL	30 mL
C16:0	46.7 ± 2.0^a^	45.2 ± 2.8^a^	42.5 ± 1.4^a^	42.2 ± 2.6^a^
C18:0	11.9 ± 1.4^a^	10.5 ± 0.4^ab^	8.6 ± 0.5^bc^	8.5 ± 0.1^c^
C18:1	17.8 ± 1.4^a^	19.8 ± 3.3^a^	23.6 ± 1.9^a^	24.0 ± 3.1^a^
C18:2	13.0 ± 0.2^c^	14.3 ± 0.5^b^	16.5 ± 0.4^a^	15.8 ± 0.2^a^
C18:3n6	4.5 ± 0.2^a^	4.5 ± 0.2^a^	4.3 ± 0.0^a^	4.3 ± 0.2^a^
C18:3n3	3.3 ± 0.5^a^	3.7 ± 0.4^a^	4.4 ± 0.6^a^	4.1 ± 0.5^a^

The values are in mean ± SD (n = 3). Different letters depict significance difference among the group according to one-way ANOVA at *p* < 0.05.

**Figure 2:**
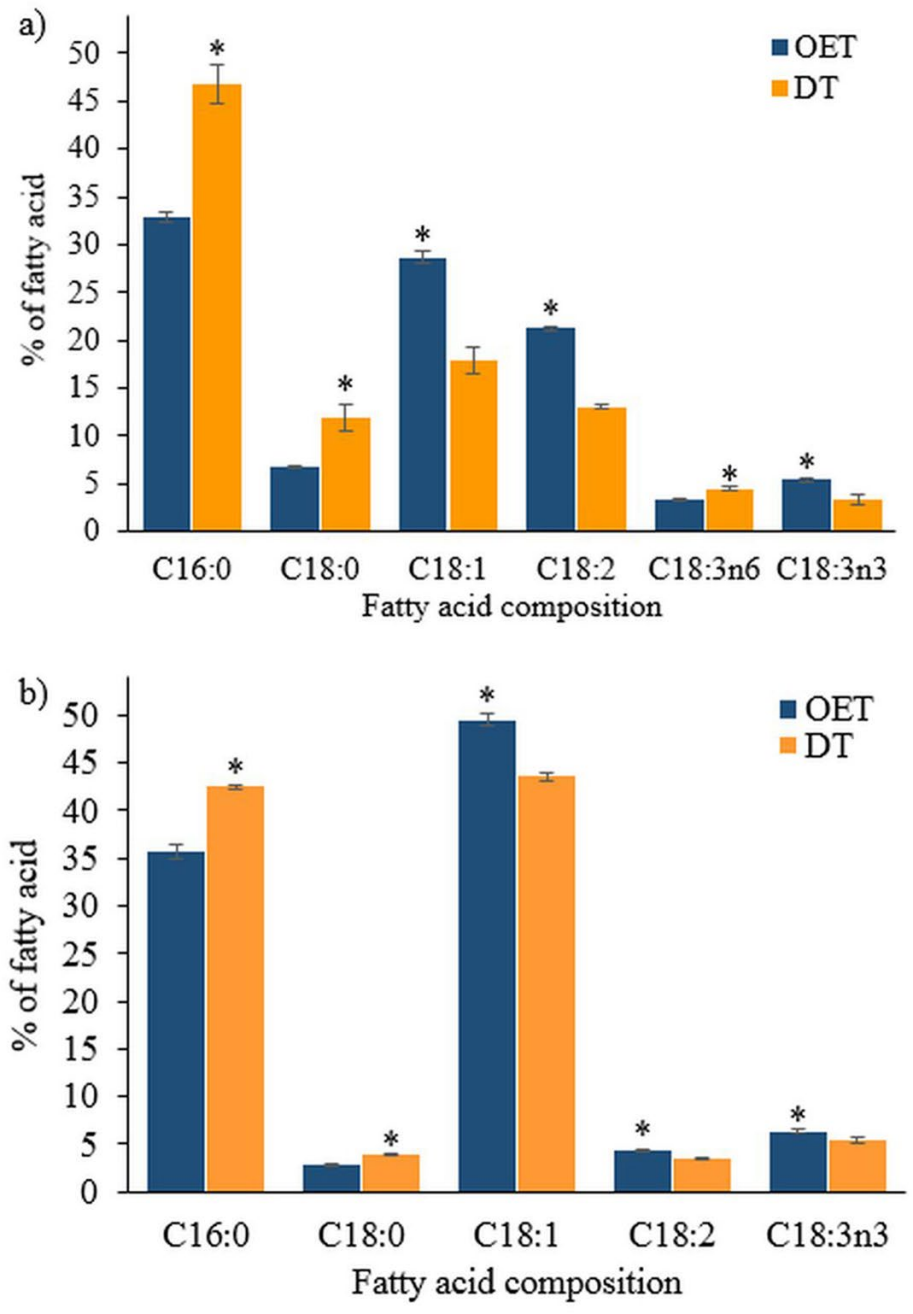
The fatty acid composition of (**a**) *C. vulgaris* UMT-M1 and (**b**) *M. gracile* SE-MC4 obtained from oil extraction-transesterification (OET) and direct-transesterification (DT) methods. The percent of fatty acid for OET and DT methods is of total oil content and total FAMEs, respectively. Asterisk (*) indicates significance higher among the same fatty acid according to *t*-test at *p* = 0.05. Data shown as the mean ± SD, n = 3.

Figure 2 depicts the comparison of major fatty acids obtained from OET and DT using 5-mL sample volume for both microalgae species. The results clearly showed the consistency of the DT method in recovering specific fatty acids. The DT method obtained significantly higher (*p* < 0.05) C16:0 and C18:0 from both species and C18:3n6 from *C. vulgaris*. For *C. vulgaris*, the C16:0 increased from 32.8 to 46.7%, C18:0 increased from 6.8 to 11.9%, and C18:3n6 increased from 3.3 to 4.5% (Fig. 2a). As for *M. gracile*, the C16:0 increased from 35.7 to 42.5%, and C18:0 increased from 2.8 to 3.9% (Fig. 2b). On the other hand, the DT method produced consistently lower (*p* < 0.05) C18:1, C18:2 and C18:3n3 for both species. For *C. vulgaris*, the C18:1 decreased from 28.6 to 17.8%, C18:2 decreased from 21.2 to 13.0%, and C18:3n3 decreased from 5.4 to 3.3% (Fig. 2a). As for *M. gracile*, the C18:1 decreased from 49.5 to 43.5%, C18:2 decreased from 4.3 to 3.4%, and C18:3n3 decreased from 6.3 to 5.4% (Fig. 2b).

There are several lipid classes contained within the microalgal cell and organelle membranes, which include neutral lipids, free fatty acids and polar lipids such as glycolipids and phospholipids^33, 36, 37^. The neutral lipids or mainly triacylglycerols (TAGs) of microalgae plays their role as storage lipids as a means for carbon and energy reserves^38^. According to Goold et al.^39^, the lipid droplet of nitrogen-starved *Chlamydomonas reinhardtii* cells that contained 80–90% TAGs and about 5–10% of polar lipids, which form a monolayer surrounding the neutral lipid core of lipid droplets. The fatty acid composition of microalgal TAGs differs greatly depending on species and culture conditions. In general, it contains fatty acids with chain-length ranging from 12 to 22 carbons with different degree of unsaturation^40^. While the polar lipids mainly consist of phospholipids and glycolipids that are important constituents of the cells and organelle membranes^8, 39, 40^. In this current study, the OET method uses hexane to extract the neutral lipids (mainly the storage TAGs) from microalgal dry biomass and subsequently the extracted lipid was transesterified into FAMEs for GC-FID analysis. Hexane (C_6_H_14_) is an alkane of six carbon atoms and serves as a non-polar solvent. It has been widely used in industries and laboratories to extract vegetable oils from crops, due to the solubility of the neutral lipids in this non-polar solvent^8^. On the other hand, the DT method used was similar to the transesterification step for OET method, but the oil sample was replaced with microalgae fresh biomass. Firstly, the fresh biomass underwent saponification when sodium hydroxide in methanol was added and the solution was boiled to saponify the oil into free fatty acid salts (soaps). These fatty acids were then transesterified to FAME with the aid of catalyst boron triflouride in methanol. The transesterification process took place where the free fatty acids, TAGs and other lipid classes (e.g. glycolipids and phospholipids) from the whole cells were converted to FAMEs for GC-FID analysis.

Therefore, the differences in fatty acid composition obtained from the OET and DT methods could serve as a tool to study fatty acid deposition dynamic between the TAGs and the polar lipids in the cell and organelle membranes. As shown in Fig. 2, DT method produced significantly higher amounts of C16:0 and C18:0 from both *C. vulgaris* and *M. gracile* which may imply the important role fatty acids have under nitrogen deficiency (BBM supplemented with only 25% of its original sodium nitrate concentration) culture condition. A previous study showed that the polar lipids of *C. reinhardtii*, *C. vulgaris* UTEX 265 and *Nannochloropsis* sp. constituted a larger amount (21.7–25.8%) of C16:0^41^. In addition, the phosphatidylcholine (PC) and phosphatidylethanolamine (PE) of *C. vulgaris* was also found to constitute C16:0 of between 34.5 and 45.5% under high- and low-CO_2_ culture conditions^42^. Therefore, the DT method not only transesterifies the storage lipids (TAGs), but also transesterifies the polar lipids inside the cells membrane and organelles membrane into FAMEs.

Furthermore, the higher recovery of C16:0 and C18:0 from both *C. vu*lgaris and *M. gracile* (Fig. 2) with DT method could be an added value for biodiesel application as higher saturated fatty acids content would contribute to higher cetane number (CN)^43^. The CN is an important measurement to determine the engine ignition delay time and combustion performance^43^. The biodiesel produced from oil producing crops such as palm and coconut^44^, as well as several microalgae from the Chlorophyta strains such as *C. vulgaris*, *Chlamydomonas* sp. and *Scenedesmus obliquus*^44^ is rich in these saturated compounds, thus rendering a high CN value in the range of 61–65^44^. These CN values are well above the minimum requirement under the European specification (UNE-EN 14214) of 51^45^. However, the DT method recovered fewer C18:1 from both microalgae species under study which could be a drawback for biodiesel application. The C18:1 has been suggested for biodiesel enrichment to improve the fuel quality due to its superior attributes such as low melting point (at − 20 °C), greater oxidative stability than that of PUFAs and CN that exceeds the minimum requirement given in major biodiesel standards^45^. Nevertheless, the fatty acid composition of microalgae can be altered through the manipulation of various culture conditions to increase the C18:1 content. For examples, nitrogen limitation^25^ and phytohormones (e.g. jasmonic acid and gibberellin) supplementation^46, 47^ in *C. vulgaris* culture, while phosphate limitation^48^ in *M. gracile* culture were found to increase the C18:1 content. However, it is elusive to find a feedstock that possesses the ‘perfect’ fatty acid composition for biodiesel conversion thus the binary blending of oils^25^ from potential feedstock could be further explored.

## Conclusions

This study demonstrated the superiority of the DT method against the OET method in recovering the total FAMEs and for fatty acid composition analysis of microalgal culture. Microalgal fresh biomass directly harvested from as little as 5 mL of the microalgal culture managed to render a 36.4 and 53.0% increase in total FAMEs production from *C. vulgaris* and *M. gracile*, respectively. Generally, both methods have their advantages, respectively, in the multidisciplinary applications. In edible oil industries such as for human consumption, pharmaceutical and nutraceutical applications, OET method is preferred as it involves the extraction of oil from microalgal dry biomass. While the DT method is favoured in biodiesel production as it eliminates the needs for dewatering, biomass drying and oil extraction processes that could further reduces the energy input, time and cost of biodiesel production. On the other hand, the small-scale and rapid DT method used in this study enables faster screening for potential lipid producing microalgae strains, as well as for real-time monitoring of FAMEs production and fatty acid profile changes during microalgae cultivation.

## Materials and methods

### Microalgae cultivation

*C. vulgaris* strain UMT-M1 and *M. gracile* (previously known as *Ankistrodesmus gracilis*) strain SE-MC4^49^ were obtained from a collection of purified microalgae stock culture at Universiti Malaysia Terengganu, Malaysia. The inoculum was initiated from a single colony taken from the stock collection and cultured into 300 mL of Bold’s Basal Medium (BBM)*.* The inoculum was then supplied with constant filtered (0.22 μm filter membrane) aeration and maintained at 24 ± 1 °C under 24 h light illustrations (~ 80 μmol m^−2^ S^−1^). The cell number was determined after one week of cultivation, and harvested for the next experiment.

The initial cell number for each experimental replicate was standardized at 5 × 10^6^ cells mL^−1^ in 300 mL of BBM (supplemented with only 25% of its original sodium nitrate concentration) prepared in a 0.5 L Erlenmeyer flask. Our previous research had shown that both *C. vulgaris* UMT-M1^50^ and *M. gracile* SE-MC4^51^ produced high amount of total oil content when the culture medium was supplemented with 25% of its original nitrate concentration. In this current study, the cultivation of both microalgae under 25% nitrate supplementation was aimed to produce reasonable high amount of total oil content for the DT and OET experiments as outlined below. This allowed sample standardization and accurate measurement and comparison of the total FAMEs produced from both methods.

The cultures were maintained at 24 ± 1 °C under 24 h light illustrations (~ 80 μmol m^−2^ S^−1^) and supplied with constant filtered (0.22 μm filter membrane) air flow. The culture growth was monitored daily until stationary growth phase was attained when cell growth reached a plateau. The cell number was determined by a Neubauer haemocytometer. The optical density was measured at a wavelength of 750 nm by spectrophotometer (Shimadzu UV-1800). The cell density was determined using the calibration curve of optical density at wavelength 750 nm (OD_750_) versus cell number. The calibration curve of optical density against cell number was drawn and the slope of the curve was used to determine the cell density of the cultures. Three microalgal culture replicates were harvested, and used for DT or conventional OET methods.

### Conventional oil extraction-transesterification (OET) procedure

At the stationary growth phase, cells in 300 mL cultures were harvested by centrifugation at 8000 rpm for 5 min. The liquid was removed while the cell pellet was washed with distilled water to remove the excess nutrients. The cell pellets were then dried at 60 °C in an oven for 24 h until consistent weight was achieved. The oil extraction procedure used was previously described by Cha et al.^25^ with modifications as outlined below. Dry biomass (X) of 0.1 g was immersed in 5 mL of concentrated HCl in a test tube and vortexes for 2 min to thoroughly wet the dry biomass. The stopper was removed and the test tube was placed in a beaker containing boiling water and boiled for 30 min, while the test tube was swirled at a 5 min interval. Subsequently, the test tube was removed from the beaker and cooled to room temperature. Hexane (12.5 mL) was added into the mixture and the test tube was stoppered and shaken vigorously for 1 min. Then, the stopper was removed and the hexane (upper layer) was transferred to a pre-dry pre-weigh 250 mL flat bottom flask (X_1_) using a pasteur pipette. The extraction was repeated twice with 7.5 mL of hexane. The combined hexane extracts were evaporated in a rotary evaporator. The flask was then dried at 60 °C until consistent weight was obtained. Finally, the flask was cooled in desiccator prior to weighing (X_2_). The total oil content was calculated using formula (1) as shown below. Fatty acid methyl esters (FAMEs) was prepared using 50 mg of the extracted oil as previously described^25^. The fatty acid composition of the FAMEs was determined using gas chromatography-flame ionization detection (GC-FID) as described in section below.

1 $${\text{Total oil content }}\left( {\% {\text{ DW}}} \right) \, = \frac{{{\text{X}}_{{2}} {-}{\text{ X}}_{{1}} }}{{\text{X}}} \times 100\%$$

### Direct transesterification (DT) of microalgal fresh/wet biomass procedure

Four different small aliquots (5, 10, 20, and 30 mL) of *C. vulgaris* fresh cells were sampled at the stationary growth phase and the cells were pelleted by centrifugation at 8000 rpm for 5 min. The supernatant was removed while the cell pellet was washed once with distilled water to remove residual nutrients.

The DT method was modified from Cha et al.^25^ The freshly harvested cell pellet was transferred, by flushing it with 5 mL of 0.5 N NaOH (in methanol), into a 250 mL flat bottom flask containing a few pieces of boiling chips. Then, the flask was attached to a Liebig Condenser and the mixture was boiled with a heating mantle for 10 min. Next, 5 mL of boron-triflouride (in 20% methanol) was added and the solution was boiled for 2 min. Subsequently, 2 mL of n-heptane was added and the mixture was further boiled for another 1 min. Finally, the flask was removed from the heating mantle and vapor was allowed to condense before removing the condenser. The resulting solution was cooled at room temperature prior to being transferred into a test tube, followed by the addition of 15 mL saturated NaCl. The test tube was stoppered and shook vigorously for 15 s for phase separation. The upper n-heptane layer was then withdrawn, filtered with a nylon syringe filter and transferred into a 1.5 mL vial prior to FAMEs analysis using GC-FID as described in “Chromatographic conditions and calculation of total FAME”.

The DT method applied on *M. gracile* culture was based-on the optimized sample volume of *C. vulgaris* culture that rendered the highest total FAMEs as described above.

### Chromatographic conditions and calculation of total FAME

FAMEs measurement was performed using gas chromatography coupled with a flame ionization detector (GC-2010 Plus, Shimadzu, Japan). The chromatographic separation of FAMEs was carried out on HP-88 capillary column (0.25 mm × 100 m, 0.2 µm) (Agilent). The injector volume and injector temperature was 1 μL and 250 °C, respectively. The split ratio for the reference standard and sample was fixed at ratio 1:50. Helium was used as the carrier gas at constant flow rate of 1.9 mL min^−1^. The flow rate for hydrogen gas and air was 40 mL min^−1^ and 450 mL min^−1^, respectively. The initial oven temperature applied was 175 °C for 10 min and then increased to 220 °C for 15 min at a rate of 3 °C min^−1^. Identification of FAMEs was done by comparing the retention time with a reference standard, the Supelco 37 Component FAMEs Mix (Sigma-Aldrich).

A five-point calibration was performed using 37 Component FAMEs Mix diluted with n-heptane. The calibration standard solutions were vortexed to ensure the homogeneity prior to GC-FID analysis. The linearity plots obtained from the calibration experiment was then applied to quantitate the total FAMEs derived from OET and DT methods. The total FAMEs obtained from the 300 mL cultures was expressed in milligrams (mg) and percentage of the total dry biomass (% DW).

### Statistical analysis

All experiments were performed in triplicates. The difference in major fatty acid composition and the total FAMEs yield of the *C. vulgaris* under volume optimization were analyzed statistically by one-way analysis of variance (ANOVA) followed by Tukey test at 5% significant level. A comparison between the OET and the optimized DT methods was carried out using *t*-test using statistical software, SPSS where statistical significance was assigned at *p* < 0.05.

## Supplementary Information

Supplementary Information
