# Supplementary material for: Enhanced fatty acid methyl esters recovery through a simple and rapid direct transesterification of freshly harvested biomass of Chlorella vulgaris and Messastrum gracile
Source: Sci Rep. 2021 Feb 1;11:2720. doi: 10.1038/s41598-021-81609-6 (PMC7851148; doi:10.1038/s41598-021-81609-6)
Supplement: Supplementary file 1 — Supplementary Information [file 41598_2021_81609_MOESM1_ESM.docx]

**Supplementary Materials**

**Enhanced fatty acid methyl esters recovery through a simple and rapid direct transesterification of freshly harvested biomass of *Chlorella vulgaris* and *Messastrum gracile***

**Author names and affiliations:**

Saw Hong Loh^1,2,^*, Mee Kee Chen^1,2^, Nur Syazana Fauzi^2,3^, Ahmad Aziz^1,2^,

Thye San Cha^1,2,^*

^1^ Faculty of Science and Marine Environment, Universiti Malaysia Terengganu, 21030 Kuala Terengganu, Terengganu, Malaysia

^2^ Satreps-Cosmos Laboratory, Central Laboratory Complex, Universiti Malaysia Terengganu, 21030 Kuala Terengganu, Terengganu, Malaysia

^3^ Institute of Marine Biotechnology, Universiti Malaysia Terengganu, 21030 Kuala Terengganu, Terengganu, Malaysia

**Correspondence to: TS Cha,*

*E-mail: cha_ts@umt.edu.my*

Tel: +609-6683394

Fax: +609-6694660

**Supplementary Data**

**Supplementary Fig. S1:** The linearity plots of some key FAME standard.
